# Hippocampal contributions to semantic memory retrieval: Strategy‐specific impairments in transient global amnesia

**DOI:** 10.1111/jnp.12430

**Published:** 2025-05-02

**Authors:** Vesile Sandikci, Anne Ebert, Annika Marzina, Michael Platten, Kristina Szabo, Carolin Hoyer

**Affiliations:** ^1^ Department of Neurology, Medical Faculty Mannheim and Mannheim Center for Translational Neurosciences (MCTN) Heidelberg University Mannheim Germany

**Keywords:** category fluency, hippocampal dysfunction, semantic memory, transient global amnesia

## Abstract

Transient global amnesia (TGA), a transient memory disorder in clinical neurology, is a unique clinical model for the study of hippocampal dysfunction and its implications for memory processes. While data are rather unequivocal concerning the relevance of the hippocampus for episodic memory, there is considerable dispute about its role for semantic memory. This study aimed at exploring how hippocampal impairment, which underlies the clinical presentation of TGA, affects semantic memory retrieval, particularly with regard to different retrieval strategies. Data from the acute and post‐acute phase of 17 TGA patients and 17 healthy controls matched on socio‐demographic factors were collected. Categorical word fluency tasks were differentiated into three retrieval strategies: first, with activation of episodic‐spatial memory content; second, with novel and flexible linking of semantic memory content and third, with activation of overlearned semantic memory content. We find that hippocampal impairment during TGA significantly restricts semantic word fluency performance, with the degree of impairment depending on the retrieval strategy used and most pronounced when flexible relinking of semantic content is required. Our results suggest an important hippocampal contribution to semantic retrieval, especially in connection with novel and flexible linking of semantic content. They may furthermore be practically relevant for the early differential diagnosis and therapy of memory disorders.

## INTRODUCTION

Declarative memory allows humans to consciously operate with representations of factual and event‐related information. Tulving (1972) proposed two components of declarative memory: semantic memory, which stores general facts and knowledge, and episodic memory encoding personal experiences and events as well as their contextual details such as time and place. Recent research indicates that these memory systems interact in both the acquisition and retrieval of information (De Brigard et al., 2022; Fang et al., 2018; Greenberg & Verfaellie, 2010; Irish & Vatansever, 2020; Renoult et al., 2019). The relevance of the hippocampus in these processes has been the subject of considerable debate: while there is a general consensus regarding its importance for episodic memory, its involvement in semantic memory remains controversial (Duff et al., 2020). Results of studies on amnestic patients with medial temporal lobe (MTL) impairments are inconsistent with regard to semantic memory performance, with findings ranging from preserved to severely impaired (Hamann & Squire, 1995; Moscovitch et al., 2006). Moreover, an increasing body of evidence indicates that the hippocampus appears to play a role in semantic retrieval as well (Cutler et al., 2019; Klooster & Duff, 2015; She et al., 2023; Solomon et al., 2019), which calls for a refined conceptualization of hippocampal contributions to semantic memory processes (Duff et al., 2020; Klooster et al., 2020; Zettersten et al., 2018). Methodological heterogeneity may be one of the factors contributing to the observed inconsistencies. Many studies use standardised tasks (e.g. naming or vocabulary knowledge) in order to assess semantic knowledge in patients diagnosed with aphasia or semantic dementia. These tasks appear to lack the level of differentiation required to detect subtler nuances of semantic dysfunction (Klooster & Duff, 2015). More complex tasks have demonstrated impaired semantic memory, particularly in terms of vocabulary depth and semantic richness, in amnestic patients. For example, Klooster and Duff (2015) observed that patients with bilateral hippocampal lesions exhibit deficiencies in identifying synonyms and collocates of words as well as in listing features of words despite sufficient familiarity with vocabulary. The implementation of a vector space model of semantic search revealed that amnestic patients produced features closer to the target word, whereas healthy controls trawled through the semantic space more widely (Cutler et al., 2019). Furthermore, Duff et al. (2009) identified significant deficits in creative linguistic expression during social interaction among patients with hippocampal damage. These findings underscore the necessity for more elaborated research into semantic retrieval. The considerable heterogeneity among amnestic patients regarding underlying conditions, the type and extent of cerebral damage coupled with relatively small sample sizes further complicates the interpretation of the findings. A more homogeneous study population may considerably facilitate the generation of clearer insights.

TGA is characterized by the abrupt onset of temporary memory impairment accompanied by severe anterograde and variable retrograde amnesia (Hoyer & Szabo, 2020). Patients are unable to form new episodic memories and frequently ask repetitive questions without recalling prior responses. The similarities between TGA and MTL amnesia led to the hypothesis that TGA represents a clinical manifestation of hippocampal dysfunction (e.g. Hodges & Oxbury, 1990). Meanwhile, neuroimaging studies have identified the presence of small uni‐ or bilateral diffusion‐weighted imaging (DWI) lesions in the hippocampus in close temporal association with an episode of TGA. The aetiology of these lesions remains uncertain; they have been proposed to represent structural correlates of amnestic deficits caused by hippocampal dysfunction (Bartsch et al., 2011). Based upon findings from animal lesion studies, studies in amnestic patients with focal MTL lesions and functional imaging studies, the crucial role of the hippocampus in the encoding and retrieval of episodic memories is undisputed. The memory impairments during TGA profoundly resemble the amnestic syndromes of patients with focal MTL lesions. Accordingly, a recent systematic review of studies in samples of at least 10 patients describes spatiotemporal disorientation, massive anterograde and variable retrograde amnesia during acute TGA (Liampas et al., 2024). Due to its transient nature, the rather uniform clinical symptomatology and the circumscribed location of the presumed pathology, the study of TGA patients provides a unique opportunity to investigate hippocampal contributions to memory processes, bypassing methodological shortcomings of lesion studies. The assessment of semantic retrieval through semantic word fluency tasks has yielded inconsistent results in patients diagnosed with TGA. Some studies have reported impairments (Eustache et al., 1997; Kessler et al., 2001); others have not (Hodges, 1994; Regard & Landis, 1984). All of these studies, however, describe single cases or very small groups of TGA patients (with a maximum of four individuals). Recent research in a larger number of patients demonstrated significant reductions in semantic word fluency performance in a sample of 16 patients with acute TGA compared to their post‐acute performance and control subjects (Sandikci et al., 2022). Differences in retrieval strategies may be one major reason for the conflicting evidence. Greenberg et al. (2009) demonstrated that patients with memory impairments in disorders affecting the MTL exhibited greater difficulties in tasks requiring the reactivation of episodic memories than with those that did not. Inferring from hippocampal activation patterns in fMRI in healthy participants contrasting with former results of studies in MTL amnestic patients, Ryan et al. (2008) deduced that retrieval from less overlearned categories may be more challenging for patients because in this context, semantic knowledge has to be used in novel ways. The present study thus aims to examine semantic word fluency in TGA patients, investigating whether hippocampal dysfunction leads to varying degrees of impairment based on retrieval strategies. It is hypothesised that acute TGA is associated with significant disruption of semantic word fluency performance, with severity depending on the type of retrieval strategy. Following recovery from acute TGA, performance is expected to normalise and not differ from that of control subjects.

## METHODS

From June 2021 to March 2024, patients presenting to the emergency department of the University Hospital Mannheim with acute amnestic syndrome were included in the study if they (a) fulfilled the diagnostic criteria of TGA (according to Hodges & Warlow, 1990), (b) were still in the acute episode exhibiting anterograde amnesia as demonstrated by a short assessment using a word learning task and (c) were native speakers of the German language. Individuals presenting with amnesia other than TGA as well as pre‐existing neurological disorders expected to result in cognitive impairment such as stroke, inflammatory or neurodegenerative diseases were excluded from the study. Similarly, those who exhibited impaired cognitive screening in the post‐acute assessment, indicated by a score under the 10th percentile on the Montreal Cognitive Assessment (MoCA; Nasreddine et al., 2005; Thomann et al., 2018), impairment of verbal‐intellectual performance in the post‐acute assessment, indicated by a score under the 10th percentile in the Verbal Comprehension Index of Wechsler Adult Intelligence Scale‐Fourth Edition (WAIS‐IV; Petermann, 2014; Wechsler, 2008) and those with ongoing clinically relevant depressive disorder, indicated by a score of more than 14 in the Beck Depression Inventory‐II (BDI‐II; Beck et al., 1996; Hautzinger et al., 2006), were excluded.

The study was approved by the Ethics Committee of the Medical Faculty Mannheim at Heidelberg University, in accordance with the ethical guidelines of the World Medical Association (Declaration of Helsinki, 6th revision, 2008). Patients provided informed written consent on the day of the episode; due to their anterograde memory deficits, a follow‐up explanation was provided on the following day, and consent was reaffirmed. Control subjects were recruited from the relatives of TGA patients and through the dissemination of flyers and online advertisements. The participants were informed about the study's objectives and methodology in written form and through direct communication with a study investigator. Neuropsychological assessments of TGA patients were conducted during the acute episode and at a 30‐day follow‐up appointment. One day after the TGA episode, BDI‐II assessment took place. MRI scans, including DWI sequences aligned parallel to the long axis of the hippocampus, were performed in the first 72 h and approximately 30 days after the TGA episode. Healthy controls underwent neuropsychological testing and MRI only once.

We developed categorical word fluency tasks to refine the assessment of retrieval strategies outlined by Greenberg et al. (2009). Three strategies were defined, each with three tested categories: (a) ‘episodic‐spatial’ (ES) with activation of episodic‐spatial memory content (items in kitchens/living rooms/bedrooms),^1^ (b) ‘novelly linked’ (NL) with novel and flexible linking of semantic memory content (items that are expensive/heavy/typically red) and (c) ‘overlearned’ (OL) with activation of overlearned semantic memory content (fruits/occupations/animals). Participants were asked to generate as many exemplars as possible in each category in 1 min. Responses were noted by the experimenter and recorded for transcription. All recorded answers were independently rated by two neuropsychologists and diverging answers were discussed. For each subcategory, specific criteria were defined for how to categorize an answer as correct or incorrect. For all categories, repetitions and synonyms were rated as incorrect. Whenever both a supercategory and a subcategory were named, the supercategory was no longer counted as correct; for example, in the OL category, animals ‘bird’ would be correct as long as no single birds (e.g. eagle, pigeon, robin) were named additionally. Regarding the NL retrieval strategy, the adverb ‘typically’ in the instructions was essential; for example, in naming items that are typically red, all items that frequently occur in other colours as well were rated as incorrect (e.g. ‘apple’ or ‘sweatshirt’). The tasks were administered in the following order: OL, ES and NL, with one category for each strategy in a randomized order. This resulted in three blocks of semantic fluency testing with intermittent testing of other cognitive functions. To ensure that the TGA patients were still in the acute phase throughout the neuropsychological examination, the presence of anterograde amnesia was repeatedly assessed using three parallel versions of the MoCA word learning task. In the learning phase, five words were read twice and patients were instructed to recall them immediately afterwards. After a 5‐min delay, free recall was tested, followed by recognition for any words that were not freely recalled (identifying the target item between two distractors). Neuropsychological testing was terminated when patients showed signs of recovery in anterograde memory function (free recall ≥1 or recognition ≥4).

### Statistical analysis

IBM SPSS Statistics, version 29, was used for statistical analysis, with an alpha level of *p* < .05. Differences between the TGA and control samples for gender, age, education, BDI‐II scores, MoCA performance and Verbal Comprehension Index were assessed using chi‐squared tests, *t*‐tests and Mann–Whitney *U*‐tests as appropriate. *T*‐tests and repeated measures ANOVAs, along with post hoc comparisons, were conducted for group comparisons of metric variables and were used to test hypotheses. Assumptions for these tests were verified using Kolmogorov–Smirnov tests, Levene tests and Mauchly tests. A two‐factor ANOVA with repeated measures on both factors *retrieval strategy* and *group* was used to compare TGA acute and TGA post‐acute. For comparisons between TGA acute and control subjects as well as TGA post‐acute and control subjects, mixed model two‐factor ANOVAs with a factor *group* and repeated measures on *retrieval strategy* were used. Due to the exploratory nature of the study, no adjustment for multiple testing was performed.

## RESULTS

Seventeen patients with TGA and 17 healthy controls participated in the study. One patient withdrew consent and was excluded. For two patients, only acute‐phase data were collected due to missed post‐acute follow‐up; however, they consented to the use of the acute‐phase data. All participants were Caucasian, native German speakers living in Germany. The mean duration of the amnestic phase was 6.69 h (SD = 2.07). The neuropsychological examination during the acute TGA episode began approximately 3 h after onset (M = 3.01 h, SD = .94). Data from 17 patients who completed both phases and 17 control subjects were included in the final analysis. None of the participants had acute depressive symptoms defined as BDI‐II > 14 (BDI‐II: M = 4.56, SD = 3.86 for patients in acute phase; M = 5.35, SD = 5.14 for patients in the post‐acute phase; M = 5.03, SD = 4.82 for control subjects), had a pre‐existing neurological disorder, showed relevant cognitive decline or reduced verbal‐intellectual ability. In addition, there were no significant differences between TGA patients and controls in terms of gender, age, education, MoCA or Verbal Comprehension Index (see Table 1 for details). MRI did not reveal tissue abnormalities in any of the participants; in 13 patients, one or more hippocampal DWI‐hyperintense lesions were found.

**TABLE 1:** Description of the study population.

	TGA *n* = 17	CG *n* = 17	*p‐*Value
Gender, male; number (%)	11 (64.7%)	9 (52.9%)	.486
Age, years; M (±SD)	63.82 (±11.52)	62.76 (±9.43)	.771
Education, years; Mdn [Q1; Q3]	13.00 [12.50–17.50]	15.00 [13.00–17.00]	.549
MoCA‐score^a^; Mdn [Q1; Q3]	28.00 [26.00–29.00]	28.00 [26.00–28.50]	.599
Verbal Comprehension Index^a^; M (±SD)	86.12 (±15.40)	92.47 (±12.08)	.190

Abbreviations: CG, control group; M, mean; Mdn, median; Q1, first quartile; Q3, third quartile; SD, standard deviation; TGA, transient global amnesia.

^a^ Assessed in post‐acute phase; for Montreal Cognitive Assessment (MoCA): maximum 30 points, for Verbal Comprehension Index: maximum 119 points.

Word fluency was assessed using three different retrieval strategies: ES, OL and NL, with three subcategories. For each strategy, a total score was calculated based on the number of correct responses across all associated subcategories. All variables met the criteria for parametric testing; ANOVAs were conducted with *retrieval strategy* (ES, NL and OL) and *group* as factors.

### TGA acute versus post‐acute

A two‐factor ANOVA with repeated measures on both *retrieval strategy* and *group* revealed a significant main effect for *retrieval strategy* (*F*(2, 32) = 89.21, *p* < .001, *η* ^2^_p_ = .85), with the fewest generated items for NL content (M = 22.97, SD = 6.88 for NL, compared to M = 45.71, SD = 11.69 for ES and M = 51.24, SD = 13.43 for OL). The *group* factor was also significant (*F*(1, 16) = 59.31, *p* < .001, *η* ^2^_p_ = .79), indicating that TGA patients performed worse in the acute phase (M = 34.53, SD = 13.32) than in the post‐acute phase (M = 45.41, SD = 17.54). A significant interaction between *retrieval strategy* and *group* (*F*(2, 32) = 3.65, *p* = .037, *η* ^2^_p_ = .19) indicated that the impairments varied by retrieval strategy. Post‐hoc *t*‐tests showed that TGA patients named significantly fewer items in the acute phase than in the post‐acute phase: 11.41 fewer for ES (*t*(16) = −6.56, *p* < .001, *d* = −1.59), 7.83 fewer for NL (*t*(16) = −6.23, *p* < .001, *d* = −1.51) and 13.41 fewer for OL (*t*(16) = −5.58, *p* < .001, *d* = −1.35).

### TGA acute versus control subjects

A mixed‐model two‐factor ANOVA with repeated measures on *retrieval strategy* but not on *group* indicated significant differences in fluency based on *retrieval strategy* (*F*(2, 64) = 224.14, *p* < .001, *η* ^2^_p_ = .88, with M = 49.41, SD = 14.43 for ES, M = 24.29, SD = 7.98 for NL and M = 52.68, SD = 13.82 for OL). The main effect for *group* was also significant (*F*(1, 32) = 27.99, *p* < .001, *η* ^2^p = .47), showing that TGA patients (M = 34.53, SD = 13.32) performed worse than control subjects (M = 49.73, SD = 18.39). The interaction of *retrieval strategy* and *group* (*F*(2, 64) = 4.26, *p* = .018, *η* ^2^_p_ = .12) revealed that performance differences varied across strategies. Post hoc *t*‐tests showed that TGA patients generated significantly fewer mentions than control subjects: 18.82 fewer for ES (*t*(32) = −5.00, *p* < .001, *d* = −1.71), 10.47 fewer for NL (*t*(26.11) = −5.05, *p* < .001, *d* = −1.73) and 16.29 fewer for OL (*t*(32) = −4.23, *p* < .001, *d* = −1.45).

### TGA post‐acute versus control subjects

To compare TGA patients in the post‐acute phase with controls, a mixed‐model two‐factor ANOVA was conducted with *group* and a repeated measures factor *retrieval strategy*. The main effect of *retrieval strategy* was significant (*F*(2, 64) = 184.09, *p* < .001, *η* ^2^_p_ = .85, with M = 55.12, SD = 12.82 for ES; M = 28.21, SD = 7.08 for NL and M = 59.38, SD = 13.77 for OL). However, neither the *group* effect (*F*(1, 32) = 1.62, *p* = .212, *η* ^2^_p_ = .05; M = 45.41, SD = 17.54 for TGA post‐acute, M = 49.73, SD = 18.39 for controls) nor the interaction between *retrieval strategy* and *group* (*F*(2, 64) = 1.16, *p* = .319, *η* ^2^_p_ = .04) was significant. These results indicate no significant difference in word fluency performance between the two groups across all retrieval strategies.

Table 2 summarizes the means and standard deviations of generated exemplars for each retrieval strategy by TGA patients in both phases and control subjects, including *p*‐values and *Cohen's d* values from post hoc comparisons.

**TABLE 2:** Means (±SD) of the exemplars generated in the semantic word fluency tasks.

	TGA acute	TGA post‐acute	CG	*p*‐Value (Cohen's *d* value)		
				Acute vs. post‐acute	Acute vs. CG	Post‐acute vs. CG
Retrieval strategy						
Episodic‐spatial	40.00 (±8.49)	51.41 (±11.85)	58.82 (±13.02)	**<.001**	**<.001**	.092
				**[−1.59]**	**[−1.71]**	[−.59]
Novelly linked	19.06 (±4.38)	26.88 (±6.76)	29.53 (±7.34)	**<.001**	**<.001**	.282
				**[−1.51]**	**[−1.73]**	[−.38]
Overlearned	44.53 (±8.43)	57.94 (±14.32)	60.82 (±13.48)	**< .001**	**< .001**	.550
				**[−1.35]**	**[−1.45]**	[−.21]

*Note*: Significant *p*‐values <.05 and corresponding Cohen's *d* values are printed in bold.

Abbreviations: CG, control group; TGA, transient global amnesia.

**FIGURE 1:**
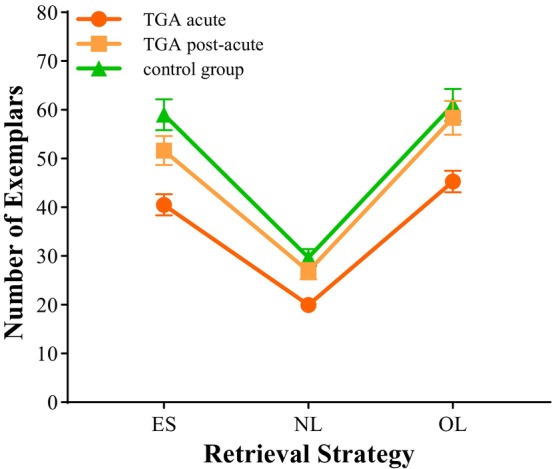
Average number of exemplars for the retrieval strategies episodic‐spatial (ES), novelly linked (NL) and overlearned (OL) with standard errors in the groups. TGA, transient global amnesia.

Figure 1 presents a line chart illustrating the average number of exemplars generated by TGA patients in both the acute and post‐acute phases and by the control subjects for each retrieval strategy.

The comparison between the acute and post‐acute phases, as well as between acute TGA patients and control subjects, seems to suggest that the loss in performance during acute TGA is least pronounced for the NL retrieval strategy. However, it is important to consider that significantly fewer mentions were made using this retrieval strategy compared to the OL and ES strategies in every group. So TGA patients named the least items when they had to link semantic memory content in a new way during TGA and after recovery, as did control subjects. This discrepancy may indicate varying task difficulties, making direct comparisons less suitable for assessing impairment in the acute phase. To further evaluate performance loss in acute TGA, we compared the acute phase performance to that in the post‐acute phase for each retrieval strategy. The performance loss was calculated by dividing post‐acute performance by acute performance, allowing for larger values to reflect a greater loss in performance. This approach provides a clearer understanding of cognitive dysfunction during acute TGA and sheds light on the importance of hippocampal function for semantic retrieval.

$$\text{Performance loss}=\frac{\text{number of exemplars generated post}-\text{acute}}{\text{number of exemplars generated acute}}$$

The *t*‐tests for dependent samples revealed that the performance loss in the ES strategy was similar to that observed in the OL strategy (*t*(16) = .17, *p* = .435, *d* = .04; M = 1.29, SD = .18 for ES, M = 1.31, SD = .22 for OL). However, the performance loss was generally greater in the NL retrieval strategy compared to the ES strategy (*t*(16) = 2.19, *p* = .022, *d* = .53; M = 1.43, SD = .28 for NL, M = 1.29, SD = .18 for ES) and the OL strategy (*t*(16) = 1.90, *p* = .038, *d* = .46; M = 1.43, SD = .28 for NL, M = 1.31, SD = .22 for OL).

**FIGURE 2:**
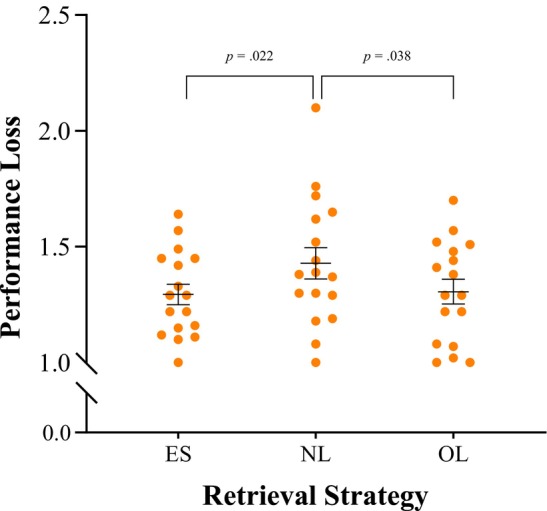
Mean performance loss for the retrieval strategies episodic‐spatial (ES), novelly linked (NL) and overlearned (OL) with standard errors of TGA patients. Larger values indicate a higher performance loss.

Figure 2 illustrates the mean performance loss in each retrieval strategy for TGA patients. As all performance quotients are higher than 1, all TGA patients showed at least some loss in each performance strategy during acute TGA. The losses are most pronounced in the NL strategy, indicating a significant decline in performance compared to the other two strategies. This suggests that the challenge of linking content novelly and flexibly contributes to greater impairment during the acute phase.

## DISCUSSION

The results indicate that word fluency performance is impaired in all three retrieval strategies, that is, ES, NL and OL, during acute TGA compared to the post‐acute phase and control subjects. This finding suggests an involvement of the hippocampus in semantic retrieval tasks. A detailed analysis of the retrieval strategies revealed that all three study groups—acute TGA patients, post‐acute patients and control subjects—generated most responses in overlearned categories, followed by episodic‐spatial retrieval strategies. The lowest performance was seen when the generation of items required novel and flexible linking of semantic memory content, highlighting the varying degrees of difficulty of these tasks. Further, TGA patients in the acute phase experienced performance losses across all conditions. Notably, there were no significant differences in performance losses between episodic‐spatial and overlearned categories. This finding contradicts the results of Greenberg et al. (2009), which suggested that patients with MTL lesions were most affected in tasks relying on episodic memory. Admittedly, we cannot be sure that all patients (and controls) reverted to the retrieval of episodic memory contents specifically in the hypothesized way. It may well be that they did not recall rooms from their episodic memory, linking the information to autobiographical memories as they were asked to by test instruction, but recalled mainly semantic memory contents (what is *typical* in kitchens, living rooms or bedrooms). We observed some TGA patients deviating from the instructed ES retrieval strategy, generating items from overlearned categories instead. For example, while naming furniture and tools you can find in kitchens, they named many food items. This suggests that participants might have accessed semantic memories linked to their own experiences, blurring the lines between episodic and semantic retrieval. On the other hand, observations of spontaneous statements during the tasks hinted towards self‐referential thinking in retrieving exemplars from overlearned semantic categories, too, activating semantic‐autobiographical and episodic content (e.g. which animals did I see in the forest, zoo… which professions have my friends and neighbours, etc.). As Greenberg et al. (2009) subsumed our strategies NL and OL in their retrieval strategy ‘neither autobiographical nor spatial’, the operationalizations are not fully compatible.

When novel and flexible combinations of semantic content were required, TGA patients demonstrated significantly pronounced performance loss in the acute phase. Ryan et al. (2008) discussed hippocampal activation in fMRI during word retrieval without reverting to episodic (or ‘autobiographic’) memory traces. They assumed that in these cases, semantic knowledge must be utilized flexibly by emphasizing relevant properties and similarities. They suggested that patients with MTL damage may perform worse in generating items from these categories compared to overlearned categories like animals or fruits. In a similar vein, our results indicate that the hippocampus is particularly engaged in tasks requiring reconnection and flexible use of information. The novel and flexible linking of semantic knowledge in word fluency tasks likely engages various executive functions, such as distinguishing essential from non‐essential features, concept formation and creative thinking. Duff et al. (2013) proposed that creativity relies on rapidly combining and recombining existing mental representations to generate new ideas. They proposed that the hippocampal system, through interaction with neocortical memory storage sites, acts as a ‘relational database’ essential for the creation, update, maintenance and comparison of mental representations utilized in declarative memory. These findings align with the Relational Memory Theory (Cohen & Eichenbaum, 1993; Eichenbaum & Cohen, 2001), which postulates that memories are the results of multidimensional relations between experience elements, highlighting the hippocampus's critical role in the comparison and integration of complex information without distinguishing between episodic and semantic content. In this regard, the interaction between the hippocampus and the prefrontal cortex (PFC) may be essential for tasks requiring cognitive flexibility. According to Morton et al. (2017), the hippocampus and medial PFC collaborate to integrate different memory traces in the creation of cognitive maps. The hippocampus reactivates old memories, while the medial PFC organizes them based on relevance. This particular conceptual framework does not differentiate between episodic and semantic memories; it interprets the formation of conceptual maps as reflecting semantic performance instead. Empirical support for these concepts comes from Solomon et al. (2019), who demonstrated that hippocampal theta oscillations (collected via depth electrodes in the MTL) reflect semantic distances between word items in memory, and the authors describe a mechanism for creating and retrieving cognitive maps in the MTL, even for non‐spatial semantic information. In TGA research, Zidda et al. (2019) asserted that the inability to form new memory traces during acute TGA goes back to reduced connectivity of the hippocampus with executive and salience networks, impairing the integration of relevant information. Further support comes from Preston and Eichenbaum (2013) who indicated that hippocampus‐PFC interactions facilitate the assimilation of new memories into existing knowledge structures, as well as Zeithamova et al. (2012), who found that functional connectivity between the hippocampus and ventromedial PFC increased with repeated exposure to nested associations. Accordingly, anatomical studies showed connections between the hippocampus and the PFC via the perirhinal cortex, the parahippocampal gyrus, and the entorhinal cortex, reinforcing the importance of their interrelations in memory processes (Lavenex et al., 2002; Suzuki & Amaral, 1994).

The results of our study suggest that the hippocampus is involved in semantic retrieval, even in the retrieval of overlearned content. While it was to be expected that the hippocampus is important to retrieve semantic content linked to episodic memory traces, we demonstrate that the hippocampus is particularly involved in the retrieval of semantic‐categorical content that requires novel and flexible linking of semantic information as well as comprehensive integration of complex episodic and semantic information.

Certain methodological limitations require mentioning. The retrieval strategies were derived from interviews and normative data from an American study (Greenberg et al., 2009). A preliminary study could have identified severely varying item difficulties connected to certain retrieval strategies. Although performance quotients were used to control for differences in item difficulty, further research should include preliminary item selection studies with healthy participants. Additionally, we observed some mingling of retrieval strategies, leading to some degree of contamination of the categories. In future studies, it may be ideal to directly ascertain whether participants followed the retrieval instructions; apparently, this is only possible in the post‐acute phase and among control subjects due to anterograde amnesia in the acute TGA phase. With regard to the assumption that TGA is a clinical model of hippocampal dysfunction, it must be considered that some studies report deficits also associated with dysfunctions in frontal, parietal and insular circuits during acute TGA (Guillery et al., 2002; Zidda et al., 2019). Thus, TGA may reflect dysfunctions in circuits incorporating the hippocampus instead of selective hippocampal dysfunction. Findings from functional imaging studies seem to point in this direction, too (Peer et al., 2014; Zidda et al., 2019).

Balancing these limitations, our study possesses several strengths: Our sample size is relatively large compared to other TGA studies with cognitive assessments during the acute phase. We ensured that the included TGA patients were suffering from anterograde amnesia until the end of testing during the acute phase. Follow‐up testing was performed by the same neuropsychologist as acute testing with the identical assessment methods in order to minimize situative‐ or researcher‐related variance. Finally, we also examined a sample of healthy controls with matching socio‐demographical characteristics.

The interactions between episodic and semantic memory systems offer significant implications for practical applications, particularly in memory training for healthy individuals, patients with MTL lesions, and those with semantic dementia. Results by Pitts et al. (2022) highlight how prior semantic knowledge can enhance episodic memory performance: older adults performed comparably to younger adults when recalling step‐by‐step instructions of everyday activities presented to them in videos when these activities were familiar to their age group. Their impaired performance in the recall of formerly unfamiliar activities suggests that the encoding and retrieval of newly learned episodic information is facilitated by the capability to link episodic information to semantic knowledge. Kan et al. (2009) demonstrated that accessing premorbid semantic knowledge facilitates episodic learning in patients with amnesia, which stresses the value of integrating individual semantic knowledge into therapeutic strategies. Savage et al. (2013) showed that in semantic dementia therapy, linking episodic memories to personally relevant terms led to significant improvements in semantic memory performance (i.e. naming).

Aligning these findings with the concepts of functional and structural neuroplasticity, targeted cognitive training, and the combination of episodic and semantic memory functions therein may foster adaptive changes in the human brain. In respect to the differential diagnosis of neurodegenerative diseases, the findings of our study could enhance the differentiation of phenotypical symptoms. For instance, assessing word fluency across different retrieval strategies (ES vs. NL) in patients with Alzheimer's dementia, semantic, and logopenic variant of primary progressive aphasia could help to facilitate earlier differentiation of clinical profiles. This, in turn, may enable the implementation of more effective interventions at earlier stages. However, further studies involving both healthy individuals and relevant patient populations are essential to validate and refine these approaches.

## CONCLUSION

The empirical findings of this work point towards hippocampal involvement in semantic retrieval, particularly in the novel and flexible linking of categorical content. This may be related to essential anatomical and functional connectivity of the hippocampus with the PFC. The hippocampus seems to play an important role in the comprehensive integration of complex information using both episodic and semantic memory traces.

## AUTHOR CONTRIBUTIONS

**Vesile Sandikci:** Conceptualization; writing – original draft; visualization; methodology; formal analysis; data curation; investigation. **Anne Ebert:** Conceptualization; methodology; writing – review and editing; data curation. **Annika Marzina:** Data curation; writing – review and editing. **Michael Platten:** Writing – review and editing. **Kristina Szabo:** Conceptualization; methodology; visualization; writing – review and editing; supervision; project administration. **Carolin Hoyer:** Conceptualization; methodology; writing – review and editing; visualization; supervision; project administration.

## CONFLICT OF INTEREST STATEMENT

All authors declare no conflicts of interest relevant to the manuscript.

## Data Availability

Anonymized data will be shared by request from any qualified investigator.

## Footnotes

^1^
Participants were explicitly asked for retrieving episodic memory contents while generating exemplars: ‘Please think of different kitchens you have been in. Within 1 min, name as many things as possible that you can find in a kitchen’.
